# SmartMal: A Service-Oriented Behavioral Malware Detection Framework for Mobile Devices

**DOI:** 10.1155/2014/101986

**Published:** 2014-08-05

**Authors:** Chao Wang, Zhizhong Wu, Xi Li, Xuehai Zhou, Aili Wang, Patrick C. K. Hung

**Affiliations:** ^1^Department of Computer Science, University of Science and Technology of China, Hefei 230027, China; ^2^School of Software Engineering, University of Science and Technology of China, Suzhou 215123, China; ^3^Faculty of Business and Information Technology, University of Ontario Institute of Technology, Oshawa, ON, Canada

## Abstract

This paper presents SmartMal—a novel service-oriented behavioral malware detection framework for vehicular and mobile devices. The highlight of SmartMal is to introduce service-oriented architecture (SOA) concepts and behavior analysis into the malware detection paradigms. The proposed framework relies on client-server architecture, the client continuously extracts various features and transfers them to the server, and the server's main task is to detect anomalies using state-of-art detection algorithms. Multiple distributed servers simultaneously analyze the feature vector using various detectors and information fusion is used to concatenate the results of detectors. We also propose a cycle-based statistical approach for mobile device anomaly detection. We accomplish this by analyzing the users' regular usage patterns. Empirical results suggest that the proposed framework and novel anomaly detection algorithm are highly effective in detecting malware on Android devices.

## 1. Introduction

Personal digital assistants (PDAs), mobile phones, and recently smartphones have evolved from simple devices into sophisticated yet compact minicomputers which can connect to a wide spectrum of networks, including the Internet and corporate intranets. Designed as open, programmable, networked devices, smartphones are susceptible to various malware threats such as viruses, Trojan horses, and worms, all of which are well known from desktop platforms. These devices enable users to access and browse the Internet, receive and send emails, and short message service (SMS), connect to other devices for exchanging/synchronizing information, and install various applications, which make these devices ideal attack targets [1].

**Figure 1:**
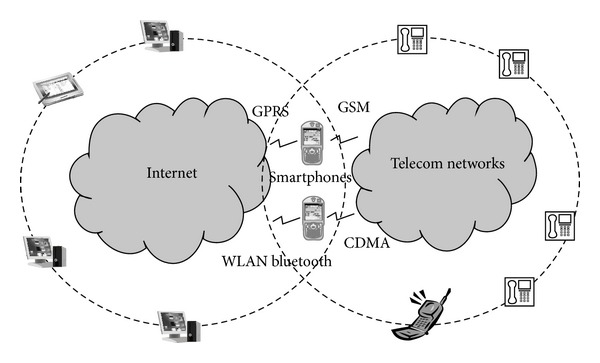
Mobile devices have become a common place for both Internet and telecom networks. They have been combined into a sound framework which allows different media to communicate with each other immediately and efficiently.

Above all, mobile devices have become popular companions in people's daily life, as is illustrated in Figure 1. It allows users to access news, entertainment, carry out research, or make purchases via e-businesses. Unfortunately, cyberspace is a double-edged sword; the new malware and viruses appearing on mobile devices have dramatically impacted the safety and security of users; this side effect of Internet access has become a serious problem. According to the Internet Filter Reviews statistics [2], the amount of malware detected is each year the double. In particular, there are at least 7.12 million smartphones that have been infected by various malware and virus.

The challenges for smartphone security are becoming very similar to those that personal computers encounter and common desktop security solutions are often being downsized to mobile devices. Unfortunately, the increasing popularity smartphones and their ability to run third-party software have also attracted the attention of virus writers [3, 4]. Malware can make a smartphone partially or fully unusable, causing unwanted billing; stealing private information, and so on. If we have the ability to detect the attack as soon as it occurs, we can stop it from doing any damage to the system or personal data. This is where an intrusion detection system comes in, there are two types of intrusion detection systems: signature-based and anomaly-based systems. Signature-based approaches can only detect existing malwares and require frequent signature updates to keep the signature database up-to-date. Signature-based systems are often used for antivirus software on desktop systems. Researchers are trying to develop anomaly based approaches which can detect unknown malwares.

Recently, behavior-based programming has been proved [5] to be an efficient way to detect abnormal utilizations to formalize requirements in the form of use cases and scenarios. It has also been introduced to the malware detection mechanism [1]. However, the behavior analysis technique is worth pursuing, it still poses significant challenge to clearly identify behaviors for distinct embedded applications.

In order to solve this problem, we will demonstrate the effectiveness of service-oriented architecture (SOA) in browser design. Traditionally, SOA provides effective measures with better flexibility and extensibility at lower cost by adopting reusable software modules. SOA can also reduce the complexity of integration and application development through uniform service description and integration interfaces. Therefore, SOA-based design is more convenient when building systems by providing a common way for interaction and communication.

By the exploration of benefits of SOA concepts, we can conclude that there are at least two significant advantages of integrating SOA concepts into malware detections. Firstly, it can greatly reduce the local workload of the detection algorithm. This feature allows users to run a light-weight client which works especially well for mobile devices, because all the processing threads will run on the servers. Secondly, the user behavior analyses, such as CPU/memory utilization, battery endurance, and network traffic flow, are located on central or distributed servers. This improves the load balancing status for global optimization. Finally, with the back-end management module (e.g., web pages), the malware information is easier to be kept up-to-date and be distributed to clients for real time synchronization.

This paper proposes service-oriented malware detection with distributed behavior analysis mechanisms for the first time, called SmartMal. The paper is extended from the previous publication at [6]. The contributions of this paper are listed as follows. The paper starts by describing the SOA-based malware with distributed detection algorithm. The abnormal messages and irregular behaviors are provided through services. Secondly, this paper proposes and realizes a behavior analysis algorithm with SOA concepts. We integrate distributed components into a hierarchical kernel model. Diverse optimizing measures are taken to exploit the battery endurance, CPU/memory utilization and network traffic flow. Experimental results are presented to demonstrate the effectiveness of SmartMal.

The rest of the paper is organized as follows. Related work and motivation are summarized in Section 2.  Section 3 discusses the architecture and main concepts of SmartMal, including architecture, client organization, and server hierarchical models.  Section 4 describes the behavior analysis model.  Section 5 demonstrates the SmartMal with a typical case: DoS attacks. Finally, conclusion and further work are presented in Section 6.

## 2. Related Work and Motivation

Safety and security problems on mobile devices have been a major focus in the past decade. In this section, we present a review on general malware detection techniques for mobile devices.

There has been a considerable amount of research into anomaly detection in computing systems and network traffic. These include statistics-based approaches, data-mining based methods, and machine learning based techniques. A wide set of anomaly detection approaches on smartphones are built from the above techniques.

Statistical-based approaches were originally used in anomaly detection on smartphones. Cheng et al. [7] propose a collaborative virus detection and alert system for smartphones where smartphones run a light-weight agent then collect and report information to a proxy. The proxy detects viruses through a statistical approach, and it keeps track of the average number of communications. Buennemeyer et al. [8] present a scheme that monitors abnormal changes of smartphones using smart batteries.

Bose et al. [9] propose a novel behavior-based detection framework for smartphones. The behavior signatures are constructed at run time by monitoring the events and API calls via Proxy DLL. They use support vector machines (SVMs) to train a classifier from normal and malicious data. The evaluation results show that the scheme can identify current mobile malwares with more than 96% accuracy. A distributed SVM algorithm is presented in [10]; with the distributed scheme, the participating clients perform their computation in parallel and update the support vectors simultaneously, so the overhead of machine learning algorithm is efficiently decreased.

Schmidt et al. [11] present programs that monitor smartphones running Symbian and Windows Mobile OS. They demonstrate that only a few features are needed to achieve acceptable detection performance. Machine learning methods, like artificial immune system (AIS) and self-organizing maps (SOM), are applied to detect abnormal behavior on remote server, and they proposed an algorithm called linear prediction to detect change by checking four predecessors of a chosen feature. In [12], they present a novel approach to detect malware, where function calls are extracted from binaries. The centroid machine classifies an executable via clustering, in which each cluster is defined by a centroid. That is, a binary is classified as malicious if it is closer to malicious cluster, naming the distance metric as Euclidean distance.

Game theory has been introduced into the anomaly detection area of mobile phone. Shabtai et al. [1] propose a light-weight malware detection system for Android smartphone, and they developed four malicious applications for experiment. Several usual classification algorithms and feature selection algorithms are evaluated to find the best performance in these detection systems. Alpcan et al. [13] present a novel probabilistic diffusion scheme for anomaly detection based on mobile device usage patterns. The scheme models the normal behavior and their features as a bipartite graph, which constitutes the basis for the stochastic diffusion process. In the stochastic diffusion algorithm, the Kullback-Leibler divergence is used to measure the distance between the distributions. uCLAVS [14] is a web service-oriented ontology framework for malware and intrusion detection. uCLAVS is based on the idea that the files analysis can improve their performance if they are moved to the network instead of running on every host. It enables each process to enter the system files, send them to the network, and then to decide whether they are executed according to the threat delivered report. Reference [15] proposes a model to reduce on-device CPU, memory, and power resources whereby mobile antivirus functionality is moved to an off-device network service employing multiple virtualized malware detection engines. TaintDroid [16] is a system-wide dynamic taint tracking and analysis system capable of simultaneously tracking multiple sources of sensitive data.

Meanwhile, programming behavior [5] is a new mechanism that has been integrated into the malware detection approaches [1, 9]. However, it has been widely used in business process [17], cache optimization [18], and operating systems [19]. However, the current research programs have serious common drawbacks: (1) most of the verification operations as well as the databases are performed locally, which may cause significant security issues if the databases are hacked, and (2) the approaches of local browsers lack modularity, which will cause excessive and inefficient workloads for programmers.

This paper introduces a SOA concept for malware detection mechanisms, in order to construct a distributed malware detection framework with behavior analysis model. SOA is widely applied in software services, web services, operating systems, and so on. Various SOA frameworks have been proposed in many fields, such as chip design [20], mobile computing system [21], classroom scheduling [22], enterprise architecture [23], Internet browser [24], and electronic productions [25]. The advantages of SOA are to integrate various services and provide unified interfaces within different solutions.

In order to learn from the SOA concepts, we have also summarized the cutting-edge SOA researches. Alam et al. [17] present a behavioral attestation method for business processes. Zhang et al. [26] provide a presentation for proactively recommending services in a workflow composition process, based on service usage history. Haki and Forte [23] demonstrate that using the SOA concept into the enterprise architecture (EA) framework makes the best of the synergy existing between these two approaches. Zhou et al. [6] explore service composition and service dependency and propose an extended dependency-aware SOA model. A loosely coupled service-oriented implementation is presented in [27]. The architecture takes advantage of Octave models in creating and using prediction models. In this framework, every method is applied as an Octave script in a plug-in fashion. Achbany et al. [28] present an allocation method of services to tasks, but the algorithm is not applied in realistic systems. In conclusion, since SOA has the ability for software across organizations and network boundaries to collaborate efficiently, it has been widely employed in aspects of research areas to facilitate researchers.

Although there is a lot of research works related to SOA and malware detection for mobile devices platform, respectively, there is only a few studies on integrating SOA and malware detection in order to construct a service-oriented abnormal behavior analysis framework.

To utilize SOA architecture's benefits, this paper presents a services-oriented malware detection and authentication mechanisms at the server side. All clients send requests to the web servers at run time in order to obtain a list of malware behaviors. This paper is extended from previous work at [29]. The paper proposes a distributed malware detection framework with the following features:

1. a light-weight profiling and information collection application on mobile devices to record all the normal and irregular behaviors;
2. a malware detection and abnormal behavior mechanism as separate modules;
3. a set of system behavior analysis schemes which integrate CPU/memory utilization, battery endurance, and network traffic flow.

## 3. SOA Architecture Model

**Figure 2:**
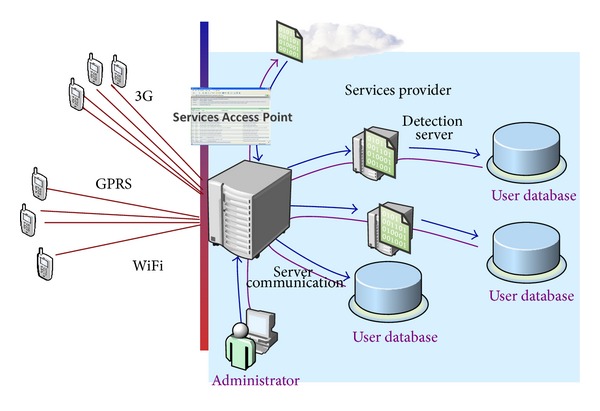
Architecture framework for SmartMal.

The concept of this paper is to apply SOA concepts into malware detection framework design. By integrating remote irregular and behavior analysis, the aim is to design an integrated client-server application with abnormal behavior maintenance. The architecture framework for SmartMal is illustrated in Figure 2. The system runs at client-server mode, of which the applications running on each client mobile device are in charge of keeping the record of the smartphones and collecting abnormal information. The selected abnormal information which is represented as vectors and extensible markup language (XML) messages are sent to the remote servers via general packet radio service (GPRS), 3G, or WiFi networks. Also the servers are distributed in one communication server and multiple malware detection servers. The communication server is responsible for exchanging messages with client users. The communication data are mainly through web services, in which data are packaged in certain data formats such as XML and JSON. After abnormal information is received, the communication server should forward the message to a specific server, in according to the client ID and system load balancing status. The detection algorithm running on the distributed servers will identify and return the results to major servers. The information will be stored in database and alerted to corresponding terminal devices when an attack or abnormal message occurs.

The SmartMal architecture provides a set of administrative control web pages. Once the data is updated, the new information will be pushed into communication servers and user clients simultaneously.

To manage the massive mobile data, the architecture maintains a* global status stable* that records the current server traffic amount data. Each server has a unique* tag*, while the major server has the smallest* tag*. The major server is elected from all the candidate servers, under the election algorithm illustrated in Algorithm 1. Denoted *S* indicates all the candidate server set, and the size of the set is *n*, represented as tag_*j*_, *j* ∈ [1, *n*].

### 3.1. Client Architecture for Mobile Devices

The Client's main function is to extract abnormal features as follows.

(*1) Feature Extractor.* This is the main module of the client. All the features are extracted through APIs provided by the Android application framework or information read from the Linux kernel. The collected features are clustered into three primary levels: Linux Kernel level (e.g., CPU, RAM, etc.), application level (e.g., messages, calling, etc.), and user behavior level. The user behavior level includes significant features that can reflect the user behavior, such as the screen on/off and the key pressed frequency. The feature extracting frequency is controlled by a timer whose value can be changed by user with the default value of 30 seconds. A total of 29 features are collected during every extracting, and the vector data structure is used to store features. As the data size of each transmission is very small (less than 200 bytes), compression mechanisms may not be able to achieve efficient performance.

(*2) Communication Module.* This module sends feature vectors to remote servers and receives anomaly alerts from the servers if the features are detected as anomaly. If the client is connected to the server for the first time, the communication module will request registration with the unique international mobile equipment identity (IMEI) of the smartphones.

(*3) Graphical User Interface (GUI).* This module provides the users with a mean to configure client parameters, such as the value of extractor timer and server IP address.

### 3.2. Anomaly Detection Server

The anomaly detection server's major task is to classify the feature vectors as normal or abnormal. The components include the following.

(*1) Database.* MySQL is used to store massive feature vectors with classValue (normal or anomaly). Database interfaces are provided for various operations. In the database, a total_table relation includes all vector information, and each detector corresponds to a detector_table, while the primary key is extract_time and phone_tag. All newly received feature vectors were stored in total_table. For each feature vector, it was assigned to the corresponding detection server according to the phone_tag and processing history. If the client was newly registered, the vector was assigned to the lowest load server.

(*2) Detecting Module.* This is the major module of the detection server, and complex detecting algorithms are implemented here. It consists of several detectors with a detector manager. Each detector is corresponding to a classification algorithm which distinguishes between normal and abnormal feature vectors, such as J48 Detector implemented with the C4.5 decision-tree algorithm. When new feature vectors come, each detector fetches the set of vectors from detector_table, builds the classifier (if it does not exist), and classifies the feature vectors. Then, the detector manager gives out the final results by integrating all detectors' results and stores the results into total_table and detector_table. The detector manager can also configure the parameters of detectors.

(*3) Communication Module.* This module communicates with the client and deals with various requests and messages. The module passes received feature vectors to detecting module, and if the vector is detected as anomaly the module will send an anomaly alert to the clients.

(*4) Client Manager.* When a new client requests for registration, this module will register the client with the IMEI.

(*5) GUI.* The server's GUI configures database and visualizes current detectors and connected clients.

### 3.3. Service-Oriented Hierarchical Model

**Figure 3:**
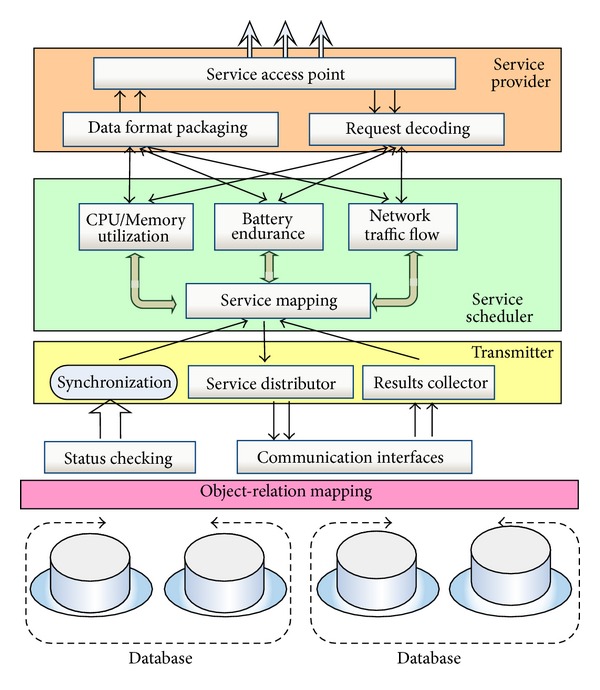
Hierarchical layer of servers.

Figure 3 presents the hierarchical model of distributed servers, which consist of three layers: services, service scheduler, and transmitter. The functionalities of each layer are introduced as follows.

First of all, services provider provides service access points (SAPs) to clients. Each SAP is in charge of one specific kind of service. All the SAPs are provided with a data format packaging mechanism. When a request arrives, the SAP first decodes the target request and then identifies which service is requested. Then, the specified request will be sent to service scheduler.

Second, services scheduler is in charge of service scheduling and mapping. Each Internet request may include several service requests. Therefore, if more than one servant is available, then each service request must be mapped and scheduled to a certain servant according to the system's load balancing status.

Finally, transmitter dispatches the subtasks to different servants for execution. After the task is completed, the results are collected by transmitter.

With respect to the period-like features of 3G/GPRS/WiFi client modules, SmartMal server provides three services for demonstration: CPU/memory utilization, battery endurance, and network traffic flow. From the exploration of the state-of-the-art studies, it is quite common that the malware applications will either drag down the network flow performance, resulting in the congestion, or illegally waste the CPU/memory utilization, or the energy.

The high level services are mapped to different servants. In order to provide a feasible system for services, at least one servant for each service is integrated into the system. Each service request is transmitted to a specific servant. All the servants are managed for efficiency use. The data transmission between servant and service layer is through communication interfaces and status checking interface.

Status checking interface is responsible for providing synchronization information of diverse servants for services mapping and scheduling, such as load balancing and services bottleneck exploration.

The physical layer consists of database and object-relation (O-R) mapping mechanisms. Generally, all the analyzed irregular behaviors are stored in databases, which may be located at distributed areas. Dealing with the current relation based database models, O-R mapping methods are widely employed for object-oriented abstractions, such as Hibernate and Toplink. Benefiting from these approaches, the high-level objects are mapped to relational databases. We hereby utilize TopLink for demonstration to map the database tuples to the standard C++ classes.

### 3.4. Remarked Features

It is quite true that spelling out the requirements for a software system under development is not an easy task, and translating captured requirements into correct operational software can be even harder [5]. Many technologies (languages, modeling tools, and programming paradigms) and methodologies (agile, test-driven, and model-driven) are designed, among other things, to help address these challenges. One widely accepted practice is to formalize requirements by behavioral programming skills in the form of use cases and scenarios.

However, in realizing abnormal detection based analysis method, not all the collected behaviors are reflecting abnormal message. Therefore, it is challenging to choose the required information among the extracted set of phases or behaviors. It has been proved that the detection efficiency can be significantly improved by the refinements of degrading the dimensions and eliminating the superfluous information [11].

In this paper, we model the weight for each feature as a synthesized combination of subjective and objective weight to identify the behavior and characteristics. The weight of the synthesized weight is represented as follows:

(1) $$\begin{array}{c} w_{i}=uw_{si}+\left(1-u\right)w_{oi}. \end{array}$$

For each feature *i*, *w*_*i*_ represents the synthesized weight, *w*_*si*_ denotes the subjective weight, *w*_*oi*_ refers to objective weight, and *u* indicates the proportion of subjective weight. The major contribution of this method is to introduce the subjective weight that can leverage the default analyzed results obtained from the behaviors. We use an analytic hierarchy process (AHP) algorithm to construct a three-layer model to divide the complex strategic decision problem into different subjects aiming at multiple targets. For each subject, a fuzzy quantitative approach is employed to calculate the weight for each feature and then merge it hierarchically.

**Figure 4:**
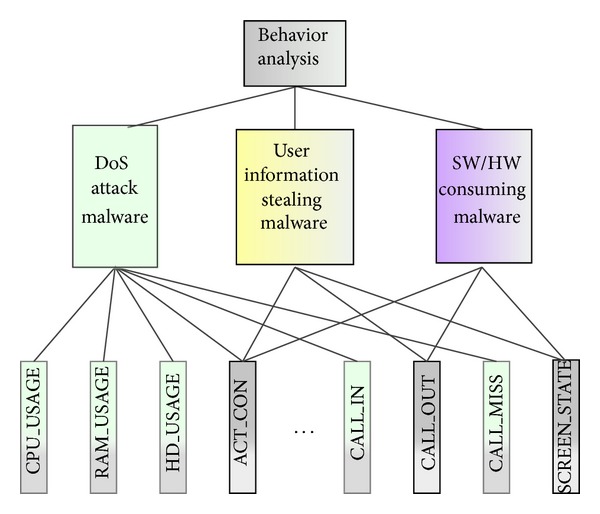
Hierarchical layers for the remarked features.

Figure 4 illustrates the hierarchical model for the remarked features that is composed of three layers. The top layer denotes the final target of behavioral analysis to identify the optimal features. In the middle layer, three classifications are listed according to different abnormal behaviors such as DoS attack malware, user information stealing malware, and irregular software/hardware resource consuming malware. Finally, all the behaviors on operating system application interfaces are reflecting the three abnormal behaviors in bottom level.

In the hierarchical analysis method, the relative weight *a*_*ij*_ represents the correlation between the *i*th element and the *j*th element. Assume that there are *n* × *m* elements in total, and then *A* = (*a*_*ij*_)_*n*×*n*_ is denoted as the correlation matrix. For the elements in the matrix, we have *a*_*ji*_ = 1/*a*_*ij*_, *a*_*ij*_ = *a*_*ij*_ · *a*_*ij*_ and *a*_*ii*_ = 1. The values and representation of different parameters are described in Table 1.

Then, we normalize the matrix *A* to matrix *Q*:

(2) Q=(qij)n×ndd,qij=aij∑k=0nakj.

Add the elements in matrix *Q* by rows to get the vector *α*:

(3) $$\begin{array}{c} \alpha ={\left(\alpha_{1},\alpha_{2},\ldots ,\alpha_{n}\right)}^{T}\;\text{in}\;\;\text{which}\;\;\alpha_{i}=\overset{n}{\underset{j=1}{\sum }}q_{ij}. \end{array}$$

The vector *α* is normalized to the weight vector *W*:

(4) W=(w1,w1,…,wn)T in  which  wi=αi∑j=1nαj.

After *W* has been calculated, we need to maintain the consistency with respect to the subjective perceptive. Consistency Index (CI) is utilized as the evaluation metrics:

(5) CI=λmax⁡−nn−1.

In (5), *λ*_max⁡_ refers to the peak value of the feature, which is derived in the following equation:

(6) $$\begin{array}{c} \lambda_{\max}=\frac{1}{n}\overset{n}{\underset{i=1}{\sum }}\frac{{\left(AW\right)}_{i}}{w_{i}}. \end{array}$$

Moreover, we also utilize consistency rate (CR) as to characterize and to model the proportion of the consistency CR = CI/RI. RI refers to random index that is the maximum value that *a*_*ij*_ is selected completely at random. Obviously, the value of CR depends on the order of the matrix *n*. The consistency is accepted only if CR < 0.1, and otherwise the correlation matrix should be leveraged until the condition is met.

After the weight for each target has been calculated, they could be moved forward to the next step, where different weight vectors are systematized as a combination. Specially, the steps to combine all the vectors are as follows.

(1) Calculate the importance for every level to the top level. This calculation process is carried out from top to bottom.
(2) Assume that there are *n*_*k*−1_ elements resided in the (*k* − 1)th level and the weight vector is calculated as
  (7) $$\begin{array}{c} W^{\left(k-1\right)}={\left(w_{1}^{\left(k-1\right)},w_{2}^{\left(k-1\right)},\ldots ,w_{n}^{\left(k-1\right)}\right)}^{T}. \end{array}$$
(3) Assume that there are *n*_*k*_ elements resided in the *k*th level and the weight vector of the impact on (*k* − 1)th level refers to
  (8) $$\begin{array}{c} p^{\left(k\right)}={\left(w_{1j}^{\left(k\right)},w_{2j}^{\left(k\right)},\ldots ,w_{nj}^{\left(k\right)}\right)}^{T}. \end{array}$$
  If the *i*th element is independent with the *j*th element, then *w*_*ij*_ = 0.
(4) From (2) and (3), we can get that the weight vector of the impact on *k*th level is
  (9) W(k)=(p1(k),p2(k),…,pn(k))W(k−1).
  After both the subjective and objective weights are evaluated, the proportion coefficient *u* can be calculated in step 1.

## 4. Behavior Analysis

To demonstrate the effectiveness of the SmartMal architecture, we have implemented a prototype application for both behavioral analysis and abnormal malware attack detection. Due to the abnormal malware detection for mobile devices, there is no acknowledged data set. For behavioral analysis, it is extremely important to select a fair and reasonable benchmark set for behavior analysis. We have selected 32 most highly ranked applications in the Android market, including 11 game applications and 21 software tools, presented in Table 2. All the software programs are installed in three mobile devices for malware detection (1 Moto Me722 handset and 2 Samsung S5830 handsets).

All the 32 applications are installed and run on the smartphones for at least one hour, during which the malware detection engines are running at back stage. The back stage engine is configured to sample the mobile device application running status every 30 seconds. After the execution is finished, there should be up to 120 items of the characterized features. For some special purposed behavior, such as battery consumption, text messages, and incoming/outgoing phone calls, it is not fair to gather the fingerprints every 30 seconds. In these application-specific scenarios, we use an accumulative value for the recent 9 intervals, each with 30 seconds. Finally, all these items are marked with normal behaviors for further comparison and detection schemes.

Alternatively, these three Android smartphones are delivered to three persons for regular daily use. Meanwhile, the back stage engine keeps on tracking the featured behavior. This period has lasted for more than 90 days.

**Figure 5:**
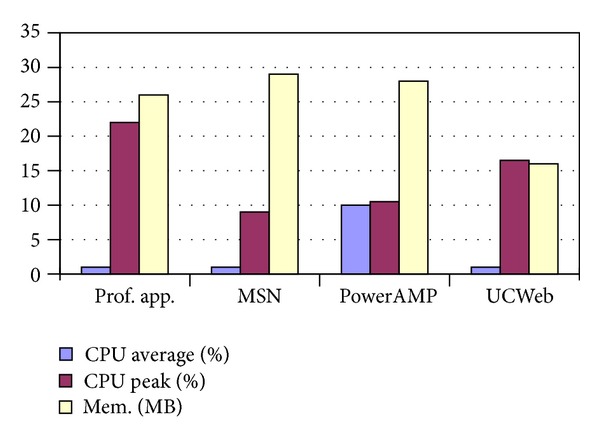
CPU/memory utilization.

In this paper, we focus on the CPU/memory utilization rate, battery consumption, and network traffic flow to analyze the workload behavior. The experimental results are illustrated in Figure 5.

### 4.1. CPU/Memory Utilization

To evaluate the CPU and memory utilization, we use* top* command in Linux operating system to observe the CPU and memory utilization of the client. Meanwhile, we have also compared the statistics for typical software applications including IM software MSN, media player PowerAMP, and UCWeb, which is illustrated in Table 3. Taking CPU average utilization rate into consideration, the profiling account only occupies less than 1% of CPU, which is ignorable. Alternatively, as our profiling application explores the feature every 30 seconds, thereby the peak utilization achieves 20%–24%, but the duration of the sample is too short to be noticed. Therefore, it does not cause any influence for user experiences. Finally, for the sake of the memory utilization, our profiling application takes up to 26 MB, which is smaller than MSN and PowerAMP applications, and, consequently, the overhead is affordable for less than 5%. Note that a general smartphone can integrate more than 512 MB internal memory.

### 4.2. Battery Endurance

One of the most serious challenges for smartphones is the power consumption and battery endurance. To analyze the behavior of how our profiling application has an impact on the battery endurance, we have evaluated the endurance trial on both scenarios, profiling online and offline, described in Table 3. On one hand, when the client application is offline, it takes approximately 525 minutes from the full battery charge 100% to 15%, while it takes 590 minutes when only 5% battery is left. On the other hand, with respect to the online profiling application, the duration only lasts for 480 minutes from 100% to 15%, while it lasts for 540 minutes from 100% to 5%.

### 4.3. Network Traffic Flow

Due to the fact that our profiling application requires Internet to transfer extracted behaviors to the major server, consequently, we also need to analyze the behavior for the network traffic flow to evaluate how our approach has an impact on the Internet utilization. We employ TrafficStats toolset provided in Android operating system to monitor the traffic flow for both single upload transaction and batch uploading process. For the sake of single uploading procedure, the packaged message should be delivered once the abnormal behavior is detected, and the uploading flow speed is 9 Kb/10 min, while the downloading speed is 4 Kb/10 min. With respect to the batch procedure, up to 10 featured messages will be batched together into a package for uploading operation. However, from the experiment, we can get a result which is exactly the same as the single uploading transaction; that is, the uploading flow speed is 9 Kb/10 min, while the downloading speed is 4 Kb/10 min. Due to the small volume for each packet, multiple messages can reside in the same package to reach a minimum size for one pack. Considering the 3G and WiFi network bandwidth, the 13 Kb/10 min network traffic flow is acceptable.

## 5. Malware Detection: A DoS Attack Case

In this section, we introduce abnormal malware detection in real DoS attack case. It is quite common that the network flow of the mobile devices statistics could be periodical. For example, the TCP SYN packages and RAB establishing/release procedures can be repeated when in a period-like manner. In particular, the mathematic idioms used in malware detection are denoted in Table 4.

### 5.1. Detection Algorithm

Generally speaking, *τ* decides the aggregation degree for the accumulated data, while distinct DoS attack behaviors on mobile networks perform different aggressive abnormal data degree. We set the minimum interval *τ*_0_ = 30 seconds, which is used to accumulate more coarse grained information, such as 1 minute, 5 minutes, 10 minutes, and 30 minutes.

Given two probability distribution for distinct time slices with same feature and intervals, the formal probability distribution can be normalized to *X* ^*τ*^(*k*_1_) and *X* ^*τ*^(*k*_2_). First, we define *L*(*k*_1_, *k*_2_) as the distance for the two probability distributions *k*_1_ and *k*_2_. Then, we need to sample the observation windows in the following two phases.

First, for the sake of a given interval *k* and observation window *W*_0_(*k*), we need to select the time slice which is most relevant to the amount of total registered mobile devices in (10) with an experimental value from 5% to 15%. Consider

(10) W0′(k)=ki∈W0(k), |N(k)−N(ki)|N(k)≤S.

Second, choose probability distribution *X* ^*τ*^(*k*) that is most relevant to *X*(*k*) from the observation window *W*_0_(*k*) to organize a sampling observation window *W*_1_(*k*).

After *W*_1_(*k*) has been identified, both internal distance *D*_1_(*k*) between *X*(*k*) and *W*_1_(*k*) and external distance *D*_*E*_(*k*) is calculated, respectively, and presented in (11) and (12). Then, the processing flow is described in Algorithm 2.  Consider

(11) $$\begin{array}{c} D_{I}\left(k\right)=\left\{L\left(k_{i,}k_{j}\right),k_{i},k_{j}\in W_{1}\left(k\right),k_{i}\neq k_{j}\right\}, \end{array}$$

(12) DE(k)={L(k,ki),ki∈W1(k)}.

### 5.2. Similarity Evaluation

To analyze whether the behavior is a normal operation or the malware attack operation, we use a Kullback-Leibler (KL) divergence based approach. Assuming that parameters *p* and *q* represent the probability distribution of two data sets, therefore, the KL divergence can be used to measure the relative entropy between the two probability distributions described in the following equation:

(13) D(p||q)=E[log⁡⁡(p(ω)q(ω))]=∑ω∈Ωp(ω)log⁡⁡(p(ω)q(ω)),

in which 0log⁡⁡(0/*q*) = 0 and *p*log⁡⁡(*p*/0) = *∞*. Moreover, the KL divergence is 0 only when *p* is equal to *q*. Since KL divergence is not a metric, we propose a revised metric to measure the distance. Consider

(14) L(p,q)=12(D(p||q)HP+D(q||p)Hq),

where *D*(*p* || *q*) and *D*(*q* || *p*) represent the KL divergence, while *H*_*p*_ and *H*_*q*_ are the entropy for *p* and *q*, respectively. In particular, the calculation of the entropy is introduced in the following equation:

(15) H(X)=(P1,P2,…,Pn)=P(xi)log⁡⁡P(xi).

Referring to (15), *X* represents the probability distribution. *P*(*x*_*i*_) indicates the probability that the source fetches *i*th signal, and we have ∑_*i*_ *P*(*x*_*i*_) = 1. Due to the calculation of *D*(*p* || *q*) which requires the additional information, *D*(*q* || *p*)/*H*_*q*_ denotes the extra workload for the calculation. To maintain the accuracy, the final distance *L* is set to the average distance of *L*(*p*, *q*) and *L*(*q*, *p*).

Referring to a probability distribution, the dimension for the two distributions can be different and an occasional case of *p*log⁡⁡(*p*/0) = *∞* may happen. In order to avoid this situation, we can choose the maximum mobile device volume as the uniform-dimensional degree, while for the insufficient distribution, we can use a signal ∈ representing 0; therefore, the situation of *p*log⁡⁡(*p*/0) = *∞* can be avoided. In this paper, we set ∈ = 10^−10^.

### 5.3. Experimental Results and Analysis

We setup a simulation platform to verify the DoS behavior detection for periodic probability distribution. The NET_SEND behavior is implemented as the TCP SYN simulation, while the back stage servers will send an abnormal malware messages demonstrating the SYN flooding attack. In order to simulate a relatively large scale experimental platform, we combine the message from 3 smartphones every 5 minutes into a 10-length chain with a 30-dimensional NET_SEND vector; then, it is normalized into the probability distribution for TCP SYN behaviors.

We run the applications on smartphones for two months continually. As one probability distribution vector includes the information collected every 5 minutes, we totally get 2 × 30 × 24 × 12 = 15480 normalized vectors.

**Figure 6:**
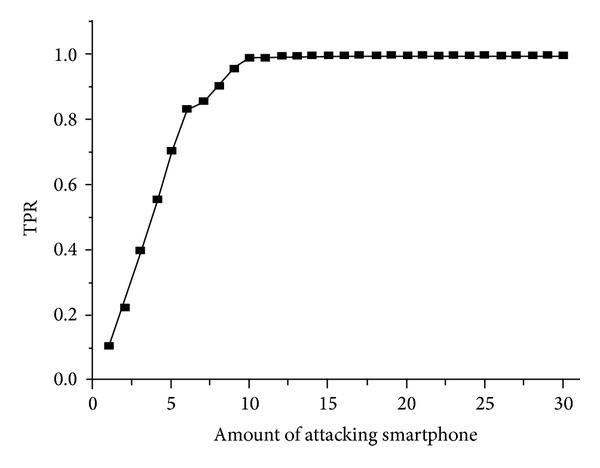
The detection accuracy for malware applications under different amounts of attacks.

Figure 6 illustrates the detection accuracy for DoS attacking malwares. The *X*-axis represents the amount of attaching smartphones, while the *Y*-axis is the detected accuracy rate for malware attacks, denoted as true positive rate (TPR). It can be easily derived that the TPR is increased with the amount of attacking smartphones. When there is only one device, the TPR is only 10%, which means more than 90% attacks failure. When the device amount is increased to 5, the TPR is also grown up to 50%. When there are more than 10 devices in total, the TPR is stable as high as 99.1%. Our approach can obtain both highly efficient and accurate results to detect all the abnormal behaviors and malware attacks.

## 6. Conclusions

This paper proposed a service-oriented malware detection framework “SmartMal,” which is the first work to combine SOA concepts with state-of-the-art behavior-based malware detection methodologies. By applying SOA into the framework, irregular behaviors can be processed in parallel servers instead of operating locally. Utilizing the distributed operation of irregular behavior analysis, SmartMal can largely reduce clients' computational complexity with great flexibility and modularity.

Moreover, as a test case, we have proposed a randomization method to defend against signaling DoS attacks on 30 cellular networks from the system-design perspective. By setting the parameter that is crucial for attacking efficiency as random distribution, the parameter is more difficult to be measured and the measured value is the maximum of the random value. The cost of launching an attack is increased enormously. Our simulation of signaling attack via RAB establishment release shows that our randomization method can achieve as high as 99.1% of the malware and irregular behaviors. The randomization method is easy and effective towards this kind of signaling attacks including paging attacks.

The initial results are promising, but there is a lot of work worth pursuing. Future works include extending the server to distributed cloud systems to achieve high throughput and integration of services. Meanwhile, we also plan to integrate the design-space exploration into the malware detection methods and behavior analysis to improve the accuracy and flexibility.

## Figures and Tables

**Algorithm 1:**
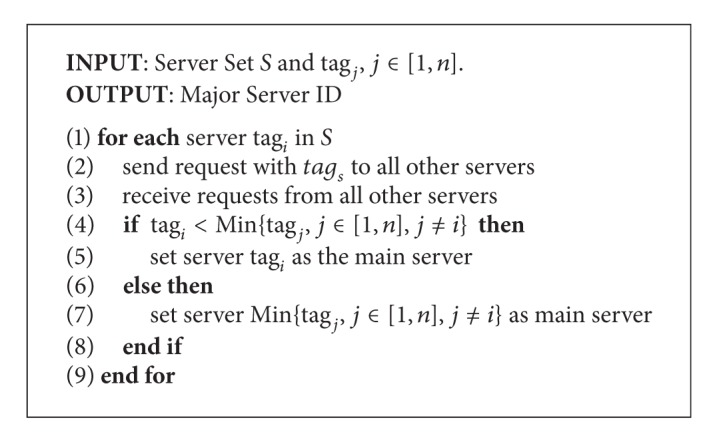
Algorithm to elect the major server.

**Algorithm 2:**
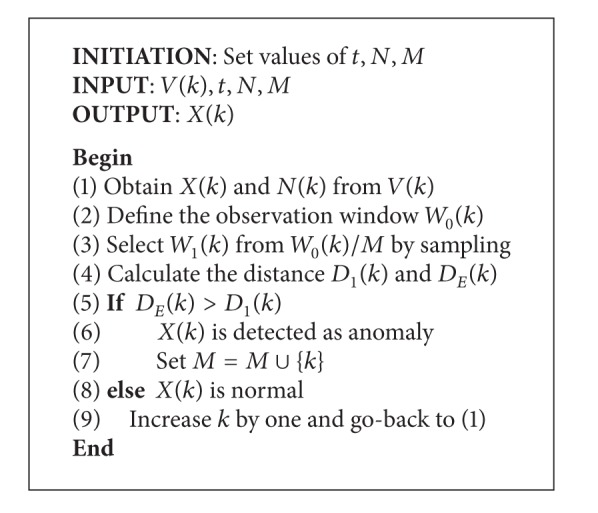
Pseudocode of abnormal behavior analysis for mobile networks.

**Table 1:** Configurations in the correlation matrix.

Value	Representation
*a*_*ij*_ = 1	*i*th element and *j*th element have the same effect
*a*_*ij*_ = 3	*i*th element is a little more important than *j*th element
*a*_*ij*_ = 5	*i*th element is important than *j*th element
*a*_*ij*_ = 7	*i*th element is much more important than *j*th element
*a*_*ij*_ = 9	*i*th element is extremely more important than *j*th element
*a*_*ij*_ = 2*n*	Superior of *i*th than *j*th element between 2*n* − 1 and 2*n* + 1

**Table 2:** Applications for profiling.

Game applications		
Fruit Ninja	Angry Birds	Can Knockdown
X Construction	Cut String	Gold Miner
Bubble Ball	Shift	Flight Chess
Sudoku	Talking Tom	

Software tool applications		

Office Suite	360 Guard	Root Explorer
King Soft	iReader	PowerAMP
Mobo Player	UCWeb	Fetion
Task Manager	MSN	Google Map
Google Music	King Reader	Mobile TV
Storm Player	Tencent QQ	Shang Mail
Sina Weibo	RenRen	Adobe Reader

**Table 3:** Battery endurance statistics.

	100% to 15%	100% to 5%
Profiling offline	525 minutes	590 minutes
Profiling online	480 minutes	540 minutes

**Table 4:** Idioms and terms defined in DoS attack cases.

*τ*	Statistics intervals
*v*_*i*_^*τ*^(*k*)	*i*th devices behavior in the interval *k*
*N* ^*τ*^(*k*)	Total amount of devices in the interval *k*
*v* ^*τ*^(*k*)	{*v*_*i*_^*τ*^(*k*), *i* = 1,2,…, *N* ^*τ*^(*k*)} the vector in interval *k*
*X* ^*τ*^(*k*)	Probability distribution for normalized *v* ^*τ*^(*k*)
*L*(*k*_1_, *k*_2_)	Similarity for *X* ^*τ*^(*k*_1_) and *X* ^*τ*^(*k*_2_)
*W*_0_(*k*)	The observation window for *X*(*k*)
*W*_1_(*k*)	The sampling observation window for *X*(*k*)
*D*_*I*_(*k*)	Internal distance for probability correlation *X*(*k*)
*D*_*E*_(*k*)	External distance for probability correlation *X*(*k*)
*M*	Abnormal probability distribution set
